# *Lycopus lucidus* Extract Attenuates Testosterone Propionate-Induced Benign Prostatic Hyperplasia in Rats

**DOI:** 10.3390/ph19091478

**Published:** 2026-09-17

**Authors:** Sueyoung An, Chan Ho Park, Enmei Gao, Jungbin Song

**Affiliations:** 1 Department of Science in Korean Medicine, Graduate School, Kyung Hee University, 26 Kyungheedae-ro, Dongdaemun-gu, Seoul 02447, Republic of Korea; asy9626@gmail.com (S.A.); cksghcksdn@gmail.com (C.H.P.); 2 ILMOK Bio Inc., 26 Kyungheedae-ro, Dongdaemun-gu, Seoul 02447, Republic of Korea; emgao@ilmokbio.com; 3 Department of Herbal Pharmacology, College of Korean Medicine, Kyung Hee University, 26 Kyungheedae-ro, Dongdaemun-gu, Seoul 02447, Republic of Korea

**Keywords:** benign prostatic hyperplasia, *Lycopus lucidus*, 5α-reductase, aromatase, androgen metabolism, testosterone, dihydrotestosterone

## Abstract

**Background/Objectives**: Benign prostatic hyperplasia (BPH) is a common age-related disorder in elderly men and a major cause of lower urinary tract symptoms. *Lycopus lucidus* Turcz. ex Benth. has been traditionally used in East Asian medicine, but its effects on BPH have not been investigated. This study evaluated the effects of *L. lucidus* extract (LLE) on testosterone propionate (TP)-induced BPH in rats and explored associated changes in androgen metabolism. **Methods**: Male Sprague-Dawley rats were orchiectomized except for the sham group and injected with TP (3 mg/kg, s.c.) for 28 days. During TP exposure, rats received oral LLE (100 or 200 mg/kg/day), finasteride (1 mg/kg/day), or vehicle. Prostate weight, prostate index, and histological changes in the ventral prostate were evaluated. Serum testosterone and dihydrotestosterone levels were measured using an enzyme-linked immunosorbent assay. The protein expression of 5α-reductase type 2, aromatase, Bax, and Bcl-2 in the ventral prostate tissue was analyzed by Western blotting. **Results**: LLE (200 mg/kg) significantly reduced prostate weight and prostate index and improved histological alterations, comparable to finasteride. LLE increased serum testosterone levels and decreased the dihydrotestosterone/testosterone ratio. Expression of 5α-reductase type 2 and aromatase in the ventral prostate tissue was significantly downregulated, while Bax and Bcl-2 expression and the Bax/Bcl-2 ratio remained unchanged. **Conclusions**: LLE attenuated TP-induced prostatic hyperplasia in rats, suggesting that modulation of androgen metabolism may contribute to its anti-hyperplastic effects. These findings support further investigation of *L. lucidus* extract in BPH.

## 1. Introduction

Benign prostatic hyperplasia (BPH) is a common age-related urological disorder characterized by hyperplasia of epithelial and stromal cells in the prostate, resulting in prostatic enlargement [1,2]. BPH causes lower urinary tract symptoms, including urinary frequency, nocturia, urgency, and weak urinary stream, which are associated with reduced quality of life and sexual dysfunction [3]. The prevalence of BPH increases markedly with age, and the global number of prevalent cases has more than doubled over the past three decades [4]. Although not malignant, progressive prostatic enlargement can lead to urinary retention, bladder dysfunction, and significant deterioration in quality of life.

The development and progression of BPH involve multiple factors, among which age-related imbalance in sex hormones is considered one of the key regulatory mechanisms underlying the disease [1,2,5]. The prostate is an androgen-dependent organ, and androgen signaling plays a critical role in the regulation of prostatic growth and function. In prostate tissue, testosterone is converted to dihydrotestosterone (DHT) by 5α-reductase, and DHT has a higher affinity for androgen receptors, leading to enhanced androgen receptor signaling and promoting prostatic cell proliferation and glandular enlargement [2,5]. In addition to androgen signaling, changes in the androgen–estrogen balance have also been suggested to contribute to the pathogenesis of BPH, particularly in association with aging [5,6,7]. Aromatase, which catalyzes the conversion of androgens to estrogens, has been implicated in local steroid metabolism involved in prostatic growth and tissue remodeling [5,8].

Given the central role of androgen metabolism in the pathogenesis of BPH, current pharmacological treatments mainly focus on inhibiting the conversion of testosterone to DHT. Representative therapeutic agents include 5α-reductase inhibitors such as finasteride and dutasteride, which reduce androgen signaling in prostate tissue, leading to decreased prostate volume and suppression of disease progression [9,10]. These agents have several limitations, including the need for prolonged administration, often for several months, adverse effects such as sexual dysfunction, and interindividual variability in therapeutic response [1,11,12]. Accordingly, increasing attention has been given to natural products and herbal medicines as alternative therapeutic candidates for BPH with potentially improved safety profiles. Representative phytotherapeutic agents include *Serenoa repens* (saw palmetto), *Prunus africana* (pygeum), and *Urtica dioica* (nettle root), which have been widely studied for the management of BPH-related lower urinary tract symptoms [13].

*Lycopus lucidus* Turcz. ex Benth., a perennial herb belonging to the Lamiaceae family, is widely distributed in East Asia. In traditional Asian medicine, the aerial part of this plant, known as Lycopi Herba, has long been used for the treatment of menstrual disorders such as amenorrhea and dysmenorrhea, which are traditionally considered to be associated with blood stasis. In traditional medicine theory, blood stasis is regarded as one of the pathological factors contributing to urinary dysfunction, and therapeutic approaches aimed at removing blood stasis have been applied in the management of related disorders, including symptoms corresponding to BPH [14]. Lycopi Herba is recognized as one of the representative medicinal herbs used for this therapeutic purpose [15]. This traditional use provided the ethnopharmacological rationale for investigating *L. lucidus* in BPH.

Phytochemical studies have shown that *L. lucidus* contains various bioactive constituents, including terpenoids such as diterpenoids and triterpenes, as well as phenolic acids, flavonoids, oligosaccharides, polysaccharides, and essential oils [16,17,18,19,20,21]. Previous pharmacological studies have demonstrated that *L. lucidus* exerts protective effects in several experimental models, including diabetic complications [22,23,24,25], metabolic disorders such as obesity and nonalcoholic fatty liver disease [26,27], and inflammatory or immune-related diseases including atopic and allergic conditions [28,29]. However, most previous studies have largely focused on metabolic or inflammatory disorders, and, to our knowledge, the effects of *L. lucidus* on BPH have not been reported.

Accordingly, we investigated the effects of *L. lucidus* extract (LLE) on testosterone propionate (TP)-induced BPH in rats. We examined its effects on prostatic enlargement, histological alterations, and androgen metabolism by evaluating serum androgen levels and the expression of 5α-reductase type 2 and aromatase in the prostate tissue. In addition, the expression of apoptosis-related proteins was examined to assess whether the anti-hyperplastic effects of LLE are associated with apoptotic signaling.

## 2. Results

### 2.1. High-Performance Liquid Chromatography (HPLC) Analysis of LLE

**Figure 1:**
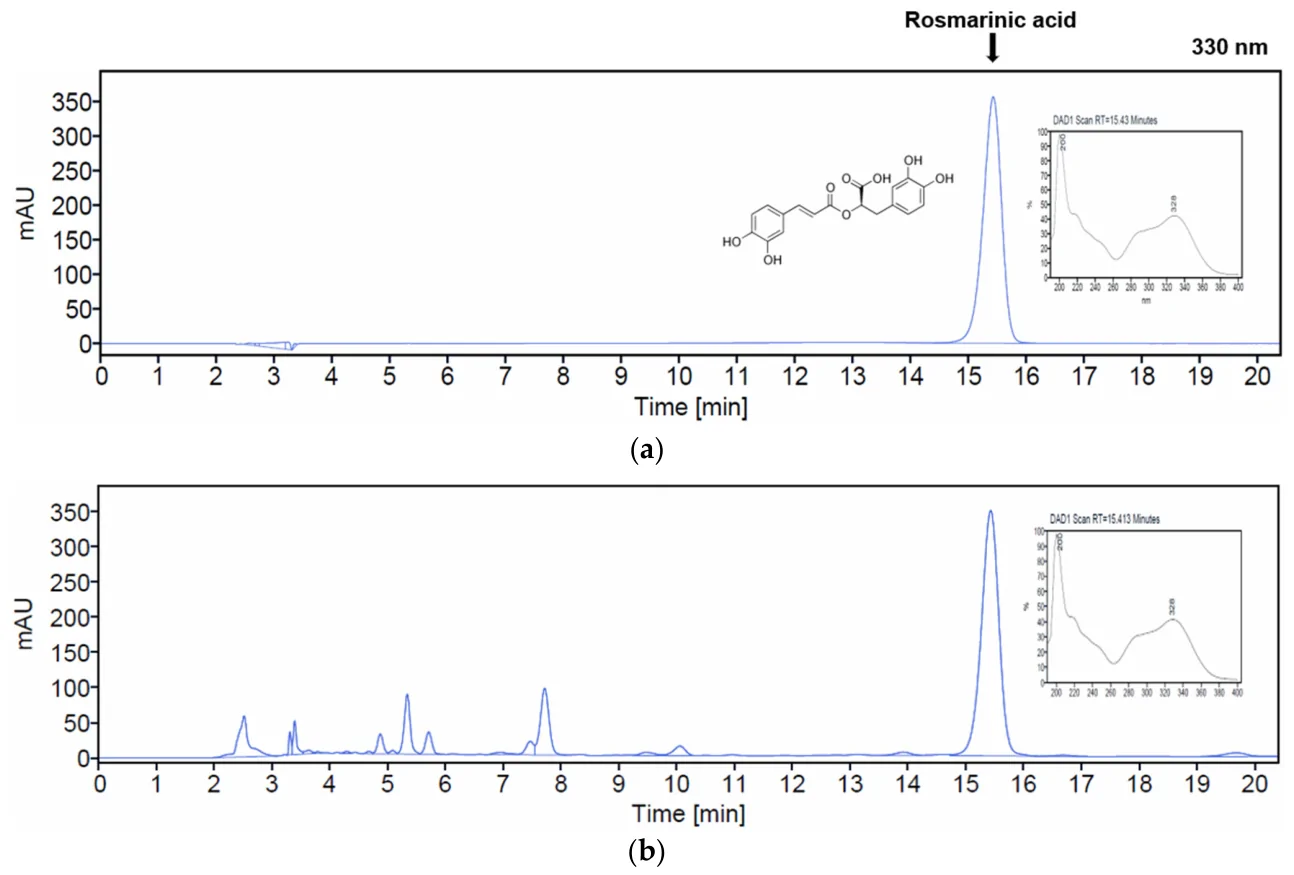
Representative high-performance liquid chromatography–diode array detection (HPLC-DAD) chromatograms of (**a**) rosmarinic acid reference standard and (**b**) *Lycopus lucidus* extract (LLE), detected at 330 nm. Insets show the corresponding UV absorption spectra.

The calibration curve for rosmarinic acid showed good linearity over the concentration range of 0.0625–1 mg/mL, with a correlation coefficient of 0.9981. The rosmarinic acid peak in LLE was detected at a retention time of approximately 15.4 min (Figure 1). Quantitative analysis showed that LLE contained 22.03 mg of rosmarinic acid per gram of extract.

### 2.2. Clinical Observations and Body Weight Changes

No mortality or clinical abnormalities were observed in any group throughout the study period. Body weight at surgery did not differ significantly among the groups (Table 1). Compared with the control group, the sham group showed significantly higher body weight on Day 1 (*p* < 0.05) and Day 29 (*p* < 0.001), as well as greater body weight gain from Day 1 to Day 29 (*p* < 0.001). Within the TP-treated groups, no significant differences from the control group were observed in body weight or body weight gain during the treatment period (Table 1).

### 2.3. Effects of LLE on Prostate Weight and Prostate Index

**Figure 2:**
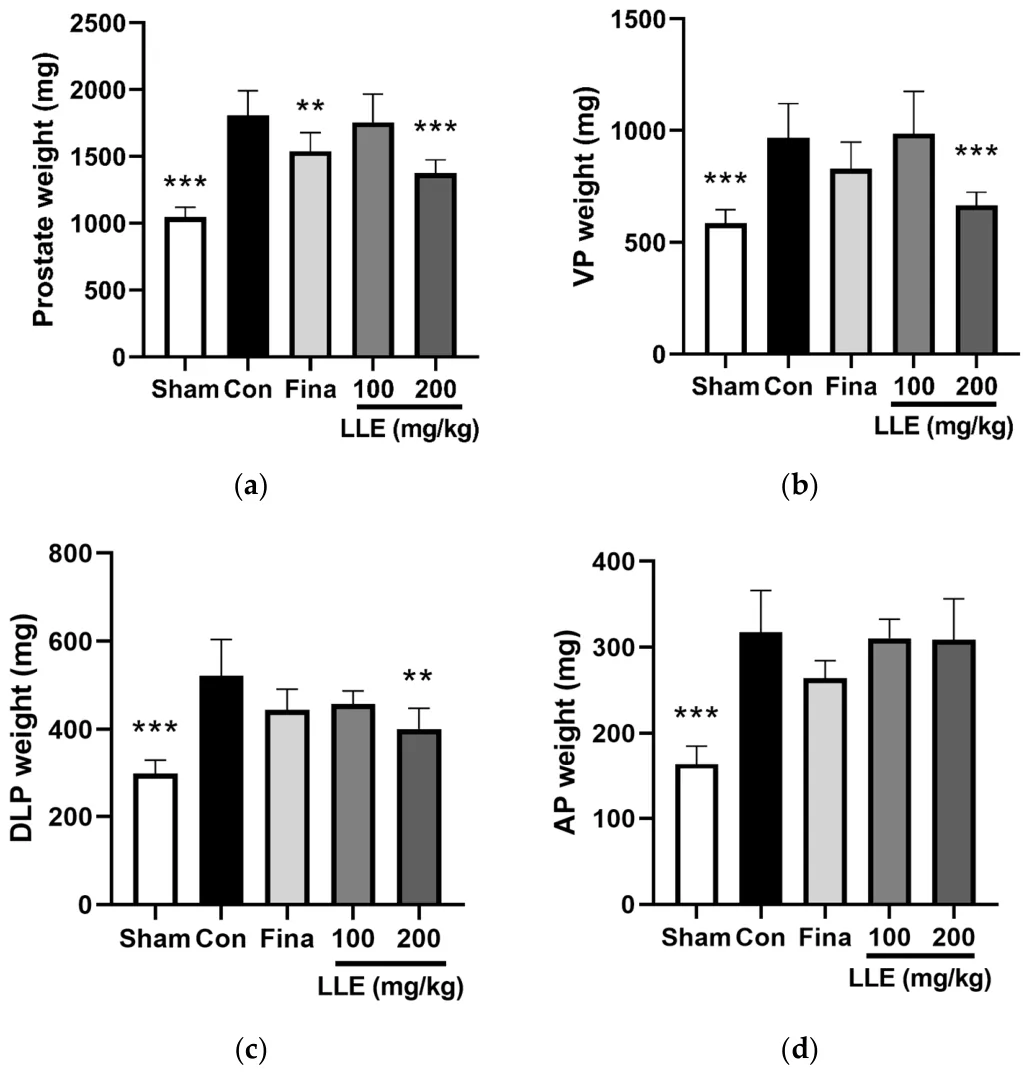
Effects of *Lycopus lucidus* extract (LLE) on prostate weight in a rat model of testosterone propionate-induced benign prostatic hyperplasia. (**a**) Total prostate weight; (**b**) ventral prostate (VP); (**c**) dorsolateral prostate (DLP); (**d**) anterior prostate (AP). ** *p* < 0.01 and *** *p* < 0.001 vs. Control (Con). Fina, finasteride 1 mg/kg.

Administration of TP for 4 weeks significantly increased total prostate weight in the control group compared with the sham group (1808.1 ± 182.6 vs. 1047.5 ± 71.3 mg, *p* < 0.001; Figure 2a). Treatment with LLE at 200 mg/kg significantly reduced prostate weight by 56.9% compared with the control group (*p* < 0.001), showing a reduction comparable to that observed with finasteride (35.4% inhibition). Lobe-specific analysis showed that LLE at 200 mg/kg significantly decreased the weights of the ventral prostate (VP) and dorsolateral prostate (DLP), whereas no significant change was observed in the anterior prostate (AP) (Figure 2b–d). LLE at 100 mg/kg did not change prostate weight.

**Figure 3:**
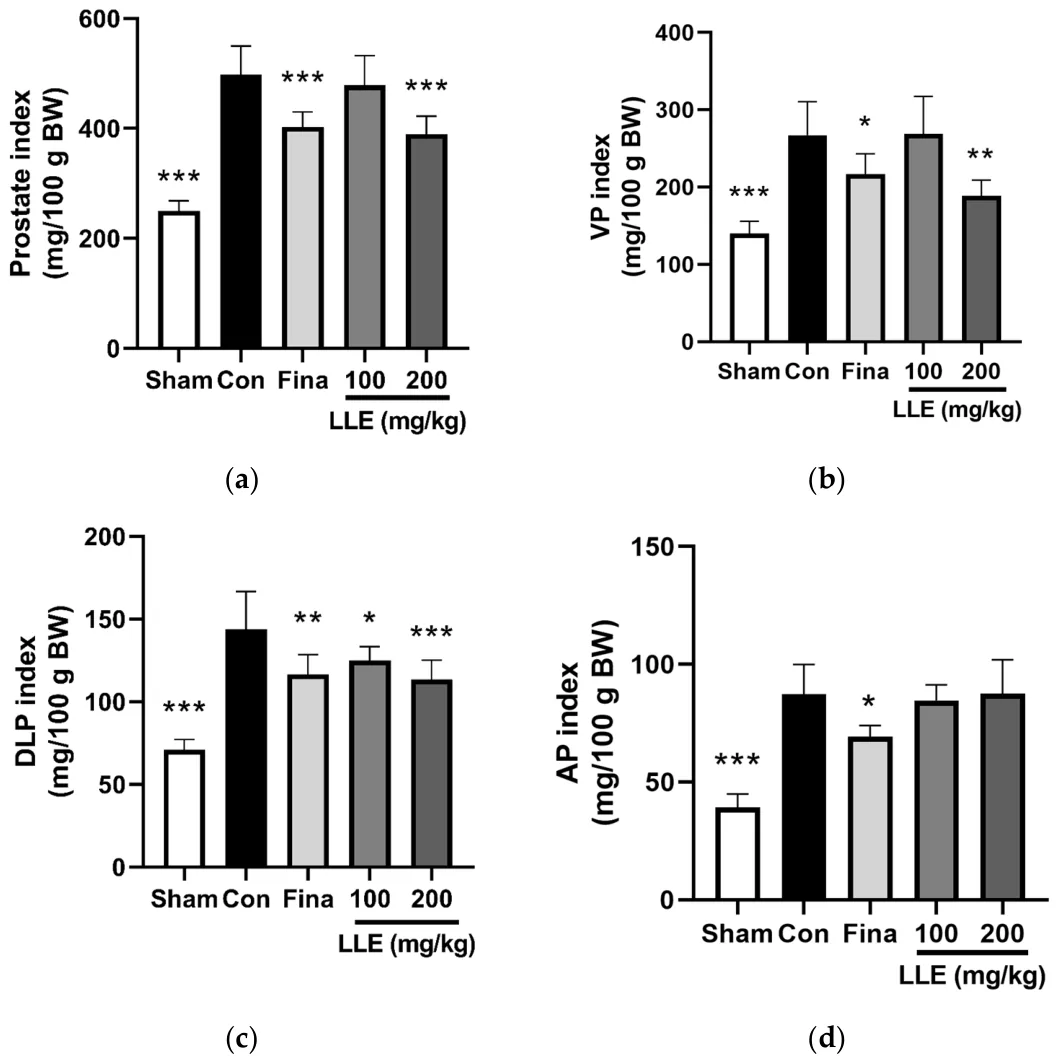
Effects of *Lycopus lucidus* extract (LLE) on prostate index in a rat model of testosterone propionate–induced benign prostatic hyperplasia. (**a**) Total prostate index; (**b**) ventral prostate (VP) index; (**c**) dorsolateral prostate (DLP) index; (**d**) anterior prostate (AP) index. * *p* < 0.05, ** *p* < 0.01, and *** *p* < 0.001 vs. Control (Con). BW, body weight; Fina, finasteride 1 mg/kg.

The total prostate index, a key measure of prostate enlargement, was significantly increased in the control group compared with the sham group (498.4 ± 51.1 vs. 250.3 ± 18.1, *p* < 0.001; Figure 3a). LLE at 200 mg/kg significantly reduced the total prostate index to 389.8 ± 32.3 (*p* < 0.001), as did finasteride (402.7 ± 27.2, *p* < 0.001). The corresponding Hedges’ *g* values versus the control group were −2.42 (95% CI, −3.61 to −1.19) for LLE at 200 mg/kg and −2.12 (95% CI, −3.32 to −0.89) for finasteride, while the comparison between the two treatment groups yielded a Hedges’ *g* of −0.41 (95% CI, −1.34 to 0.55).

Consistent with the weight data, LLE at 200 mg/kg significantly reduced the VP and DLP indices, whereas no significant change was observed in the AP (Figure 3b–d). LLE at 100 mg/kg did not affect the total prostate index but reduced the DLP index.

### 2.4. Effects of LLE on Histopathological Changes in the Ventral Prostate

**Figure 4:**
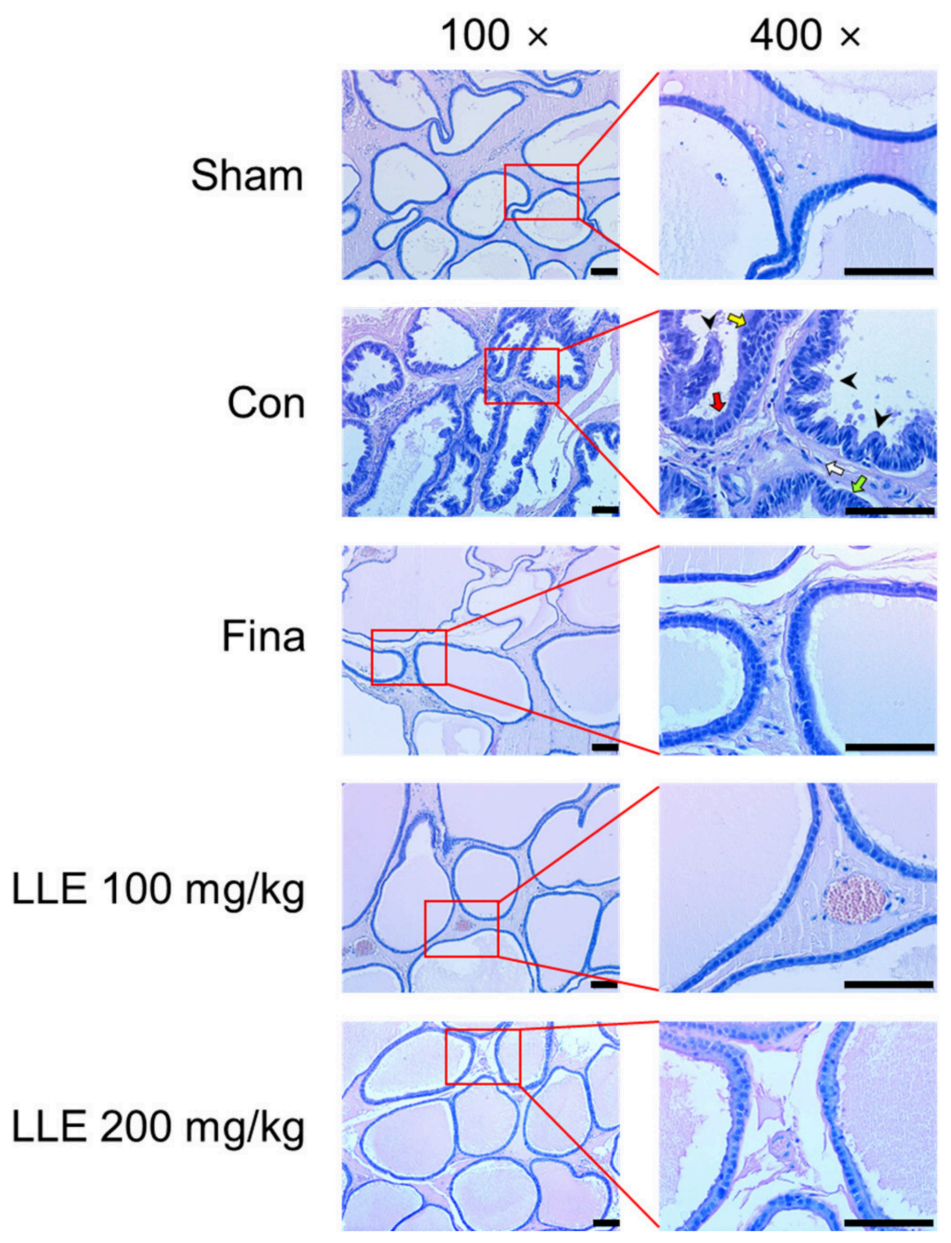
Representative hematoxylin and eosin (H&E)–stained sections of the ventral prostate. From top to bottom: sham, control (Con), finasteride 1 mg/kg (Fina), *Lycopus lucidus* extract (LLE) 100 mg/kg, and LLE 200 mg/kg. Left column, 100×; right column, 400×. Boxed areas indicate regions enlarged at higher magnification. Black arrowhead, papillary or villous protrusions; red arrow, highly columnar epithelium; yellow arrow, epithelial piling; green arrow, loss of polarity; white arrow, stromal hypercellularity. Scale bars: 100 µm.

Histological examination of the ventral prostate showed normal glandular architecture with regularly arranged acini lined by a single layer of cuboidal epithelial cells and a well-organized stromal structure in the sham group (Figure 4). In contrast, the control group exhibited marked epithelial thickening, irregular acinar arrangement, reduced luminal space, papillary or villous protrusions into the lumen, epithelial piling, loss of polarity, and increased stromal cellularity with spindle-shaped nuclei. Treatment with LLE (100 and 200 mg/kg) attenuated these histopathological changes compared with the control group.

### 2.5. Effects of LLE on Histological Score and Epithelial Thickness in the Ventral Prostate

Histological scoring analysis was performed to quantitatively evaluate histological changes in the ventral prostate. The total histological score was significantly higher in the control group than in the sham group (57.40 ± 10.25 vs. 21.87 ± 7.55, *p* < 0.001). Treatment with LLE significantly reduced the total score (34.73 ± 8.85 at 100 mg/kg and 35.33 ± 13.03 at 200 mg/kg, both *p* < 0.05) compared with the control group. Detailed scoring results for individual parameters are provided in Table 2.

**Figure 5:**
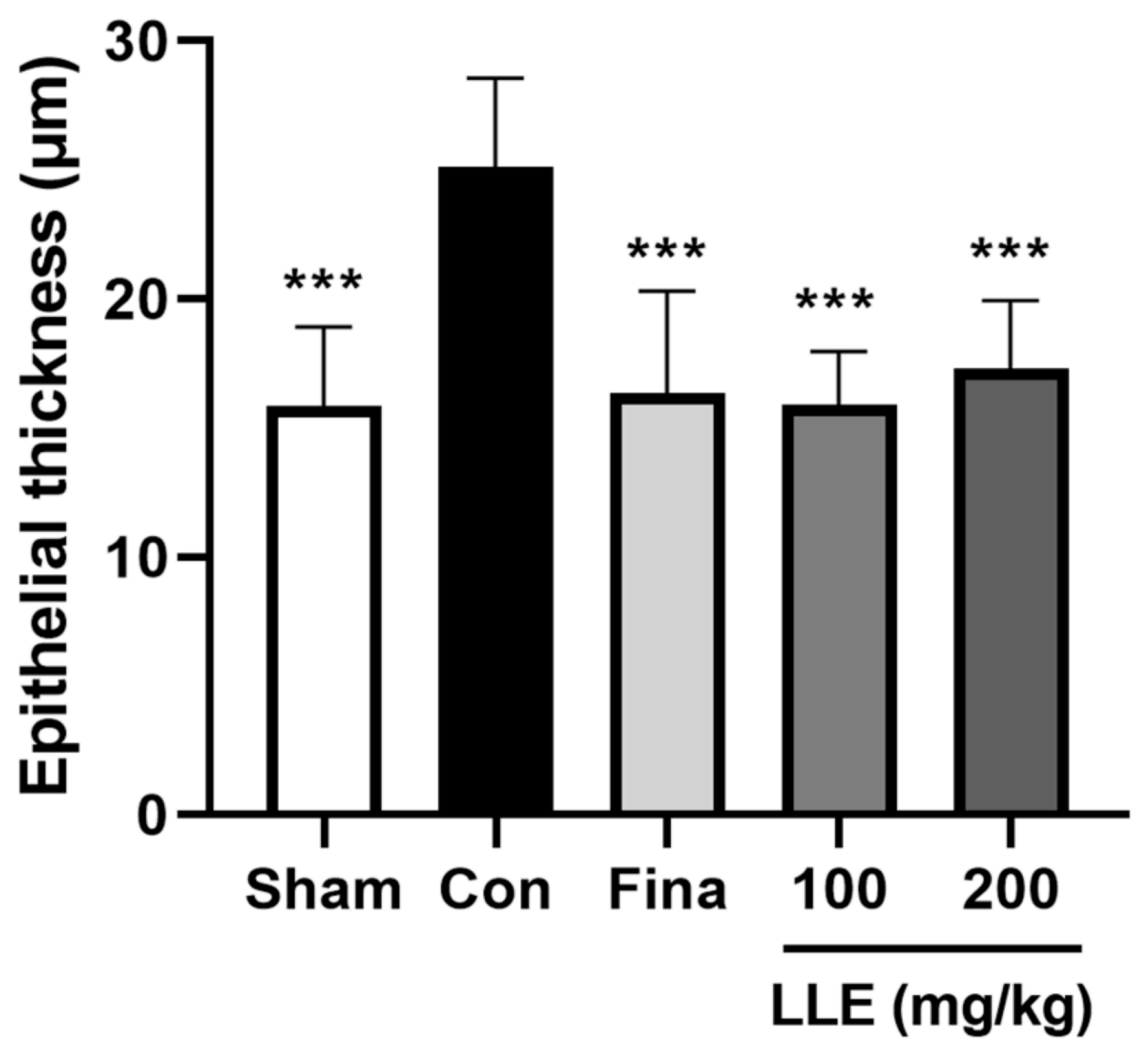
Effects of *Lycopus lucidus* extract (LLE) on epithelial thickness in the ventral prostate. *** *p* < 0.001 vs. Control (Con). Fina, finasteride 1 mg/kg.

Epithelial thickness in the ventral prostate was significantly increased in the control group compared with the sham group (25.1 ± 3.4 vs. 15.9 ± 3.1 µm, *p* < 0.001; Figure 5). Treatment with LLE (100 and 200 mg/kg) and finasteride significantly reduced epithelial thickness, restoring it to levels comparable to those of the sham group (all *p* < 0.001 vs. control).

### 2.6. Effects of LLE on Serum Androgen Levels

**Figure 6:**
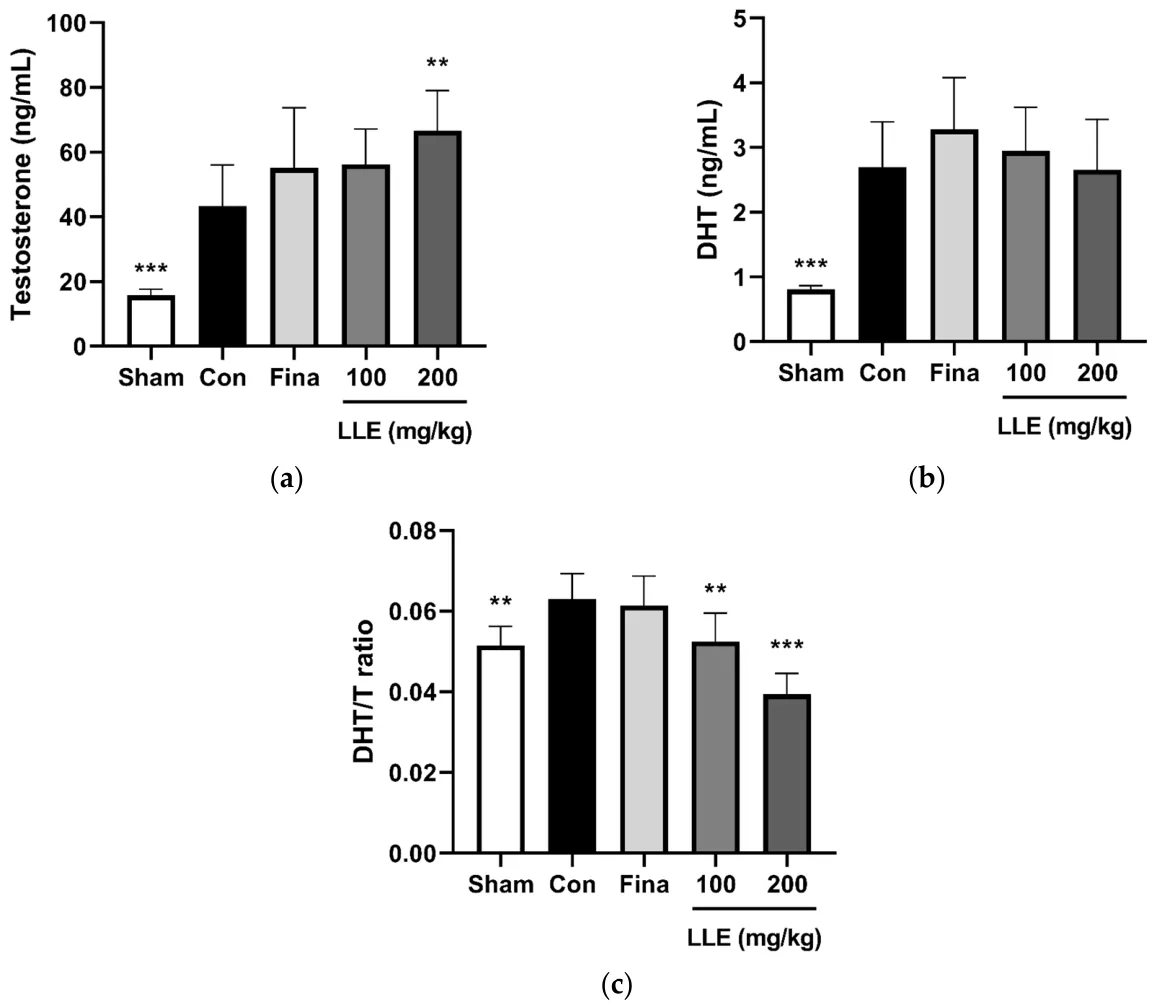
Serum levels of (**a**) testosterone, (**b**) dihydrotestosterone (DHT), and (**c**) DHT/testosterone (DHT/T) ratio. ** *p* < 0.01 and *** *p* < 0.001 vs. Control (Con). Fina, finasteride 1 mg/kg; LLE, *Lycopus lucidus* extract.

Administration of TP for 4 weeks increased serum testosterone and DHT levels in all TP-treated groups compared with the sham group (Figure 6). Serum testosterone levels were significantly higher in the LLE 200 mg/kg group than in the control group (66.6 ± 12.4 vs. 43.3 ± 12.7 ng/mL, *p* < 0.01; Figure 6a), whereas serum DHT levels showed no significant differences among the TP-treated groups (Figure 6b).

The DHT/testosterone ratio was significantly higher in the control group than in the sham group (0.063 ± 0.006 vs. 0.052 ± 0.005, *p* < 0.01; Figure 6c). Treatment with LLE significantly decreased the DHT/testosterone ratio compared with the control group in a dose-dependent manner (0.052 ± 0.007 at 100 mg/kg, *p* < 0.01; 0.039 ± 0.005 at 200 mg/kg, *p* < 0.001).

### 2.7. Effects of LLE on 5α-Reductase and Aromatase Expression in the Ventral Prostate Tissue

**Figure 7:**
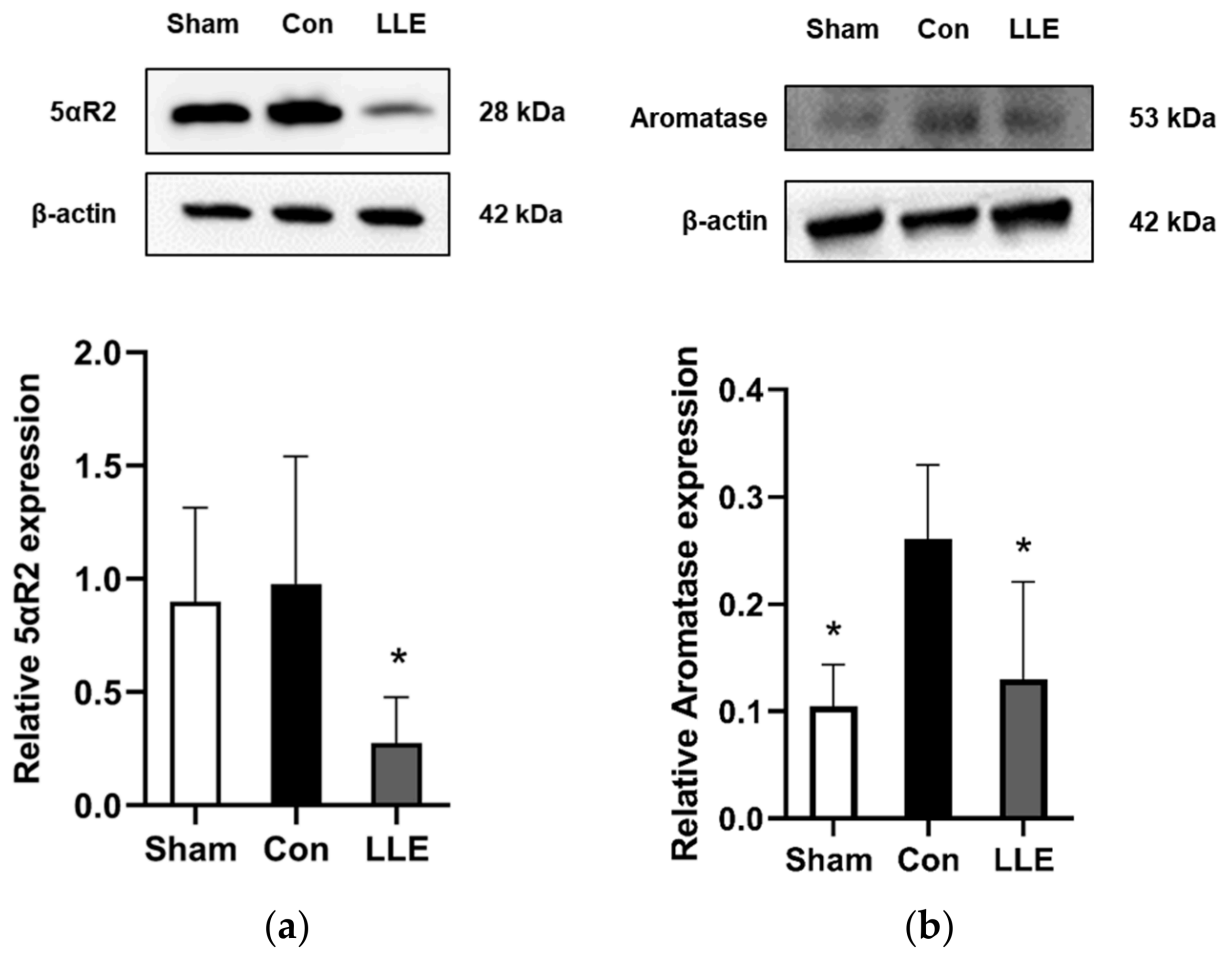
Effects of *Lycopus lucidus* extract (LLE) on prostatic 5α-reductase type 2 (5αR2) and aromatase expression. Representative Western blot images and densitometric quantification of (**a**) 5αR2 and (**b**) aromatase expression are shown. Protein expression levels were normalized to β-actin. * *p* < 0.05 vs. Control (Con). LLE, *L. lucidus* extract 200 mg/kg.

Based on its significant inhibitory effects on prostate weight and prostate index, the LLE 200 mg/kg group was selected for further mechanistic analysis. The expression of 5α-reductase type 2 in the ventral prostate tissue did not differ between the sham and control groups. LLE treatment significantly reduced 5α-reductase type 2 expression, showing an approximately 70% reduction compared with the control group (Figure 7a).

Aromatase expression in the ventral prostate tissue was increased by approximately 2.6-fold in the control group compared with the sham group and was reduced by LLE treatment to approximately half of the control level (Figure 7b).

### 2.8. Effects of LLE on Bax and Bcl-2 Expression in the Ventral Prostate Tissue

**Figure 8:**
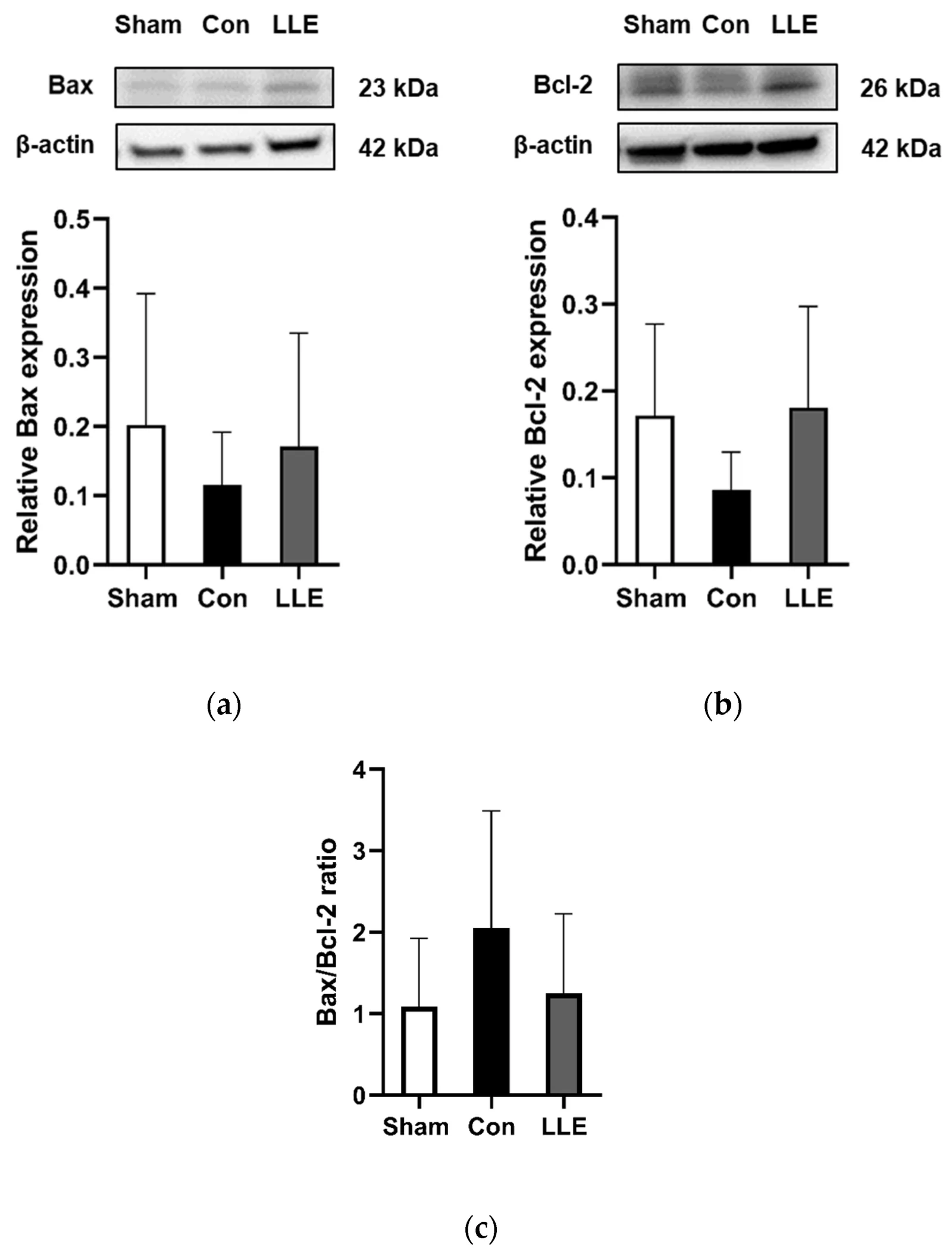
Effects of *Lycopus lucidus* extract (LLE) on prostatic apoptosis-related protein expression. Representative Western blot images and densitometric quantification of (**a**) Bax and (**b**) Bcl-2 expression are shown, together with (**c**) the Bax/Bcl-2 ratio. Protein expression levels were normalized to β-actin. Con, control; LLE, *Lycopus lucidus* extract 200 mg/kg.

The expression of Bax and Bcl-2 was examined in ventral prostate tissue to assess whether LLE affects the mitochondrial apoptosis-related regulatory axis. Bax and Bcl-2 protein levels did not differ between the sham and control groups, and LLE treatment (200 mg/kg) did not significantly change their expression (Figure 8a,b). Accordingly, the Bax/Bcl-2 ratio also remained unchanged among the groups (Figure 8c).

## 3. Discussion

In the present study, LLE attenuated TP-induced prostatic hyperplasia in rats, as demonstrated by reductions in prostate weight and prostate index together with improvements in histopathological changes. To our knowledge, this is the first experimental study to demonstrate the anti-hyperplastic effects of Lycopi Herba in a rat model of BPH. LLE also decreased the serum DHT/testosterone ratio and suppressed the expression of 5α-reductase type 2 and aromatase in prostate tissue, suggesting that its anti-hyperplastic effects are associated, at least in part, with modulation of androgen metabolism.

BPH is a multifactorial disorder in which androgen-dependent pathways play an important role in prostatic growth and disease progression [30]. Although circulating testosterone levels generally decline with age, conversion of testosterone to DHT remains a clinically relevant therapeutic target, as demonstrated by the efficacy of 5α-reductase inhibitors in reducing prostate volume and the risk of BPH progression [31,32]. The TP-induced rat model is widely used to investigate prostatic responses to androgenic stimulation and to evaluate agents that affect androgen-related pathways [33]. In this model, orchiectomy reduces variability in endogenous testicular androgen production, whereas repeated TP administration induces epithelial hyperplasia and prostate enlargement within a relatively short period [34,35]. Thus, although this model does not fully reproduce the age-related and multifactorial pathophysiology of human BPH, it provides a controlled experimental system for evaluating androgen-stimulated prostate growth.

The LLE doses of 100 and 200 mg/kg/day were selected on the basis of the recommended medicinal dose of Lycopi Herba and previous pharmacological data obtained with a comparable 70% ethanol extract of *L. lucidus* [27]. The recommended dose of Lycopi Herba is 6–12 g/day as crude herbal material [36]. Based on the extraction yield of LLE (9.84%) and body surface area–based dose conversion, 100 mg/kg/day in rats corresponds to approximately 10 g/day of crude herbal material for a 60-kg adult, which falls within this recommended range. A previous study also reported pharmacological activity of a 70% ethanol extract of *L. lucidus* at 100 and 200 mg/kg/day in mice [27]. These considerations supported the selection of 100 mg/kg/day as the reference dose and 200 mg/kg/day as a twofold higher exploratory dose.

LLE, particularly at 200 mg/kg/day, significantly inhibited TP-induced prostate enlargement, with effects of similar magnitude to those of finasteride in the ventral and dorsolateral prostate. Unlike the human prostate, which is organized into anatomical zones, the rodent prostate consists of distinct lobes that differ in 5α-reductase isozyme composition, androgen receptor expression, and responsiveness to androgenic stimulation [37,38,39,40]. The ventral and dorsolateral lobes are particularly androgen-responsive and are therefore frequently evaluated in experimental BPH models [38,40]. In the present study, LLE significantly reduced prostate weight and prostate index in these lobes, supporting its inhibitory effect on androgen-stimulated prostatic enlargement.

Histopathological findings further supported the anti-hyperplastic effects of LLE. BPH is characterized by excessive proliferation of epithelial and stromal cells, with androgen-mediated epithelial–stromal interactions contributing to disease development and progression [41,42]. LLE attenuated the characteristic hyperplastic changes induced by TP, as reflected by lower histological scores and reduced epithelial thickness. The consistency between the semi-quantitative histological assessment and quantitative morphometric analysis strengthens the evidence for the histological effects of LLE. Although semi-quantitative scoring is inherently susceptible to observer-dependent variability, this potential bias was minimized by independent blinded assessment by two investigators using predefined criteria. The histological scoring system proposed by Scolnik et al. (1994) [43] and modified by Golomb et al. (1998) [44] has not been formally validated specifically for TP-induced BPH. Nevertheless, its repeated application across experimental rat models of prostatic hyperplasia [45,46,47], together with the complementary quantitative assessment performed in this study, supports its utility for evaluating the observed histopathological changes.

An important finding of the present study was the alteration of androgen metabolism-related parameters following LLE treatment. Testosterone is converted to the more potent androgen DHT by 5α-reductase, and DHT binds the androgen receptor with greater affinity than testosterone, thereby promoting proliferation of prostatic epithelial and stromal cells [48]. The DHT/testosterone ratio has been used as an indirect indicator of 5α-reductase activity and testosterone-to-DHT conversion [49]. Although serum DHT levels did not differ significantly among the TP-treated groups, LLE decreased the serum DHT/testosterone ratio in a dose-dependent manner. In addition, serum testosterone was significantly higher in the LLE 200 mg/kg/day group than in the control group. Given that all animals in the TP-treated groups were orchiectomized and received the same dose of exogenous TP, the increase in serum testosterone is unlikely to reflect increased endogenous testicular production and may instead be related to altered metabolism or disposition of the administered testosterone. However, the mechanism underlying the increased serum testosterone remains unclear. A reduction in testosterone-to-DHT conversion is one possible explanation, but this interpretation remains inferential because serum DHT was not significantly reduced and serum hormone concentrations do not necessarily reflect the local androgen environment within the prostate.

Consistent with the changes in circulating hormone parameters, LLE significantly decreased 5α-reductase type 2 expression in the ventral prostate. 5α-reductase type 2 plays an important role in local DHT formation and androgen receptor signaling within prostatic tissue [42,50]. However, because protein expression does not necessarily reflect enzymatic activity and intraprostatic testosterone and DHT concentrations were not measured, these findings do not establish that LLE directly inhibits testosterone-to-DHT conversion. The concordant changes in the serum DHT/testosterone ratio and prostatic 5α-reductase type 2 expression support an association between LLE treatment and altered androgen metabolism that warrants further mechanistic investigation.

LLE also significantly reduced aromatase expression in the ventral prostate. Testosterone can be metabolized not only to DHT by 5α-reductase but also to estradiol by aromatase, and alterations in these pathways may affect the local androgen–estrogen balance implicated in BPH pathophysiology [7,51]. Increased 5α-reductase type 2 expression promotes local DHT formation and androgen receptor signaling [42,50], whereas increased aromatase expression enhances local estrogen production and contributes to stromal proliferation and tissue remodeling [8,52,53]. In the present study, aromatase expression was increased in the control group, consistent with previous testosterone-induced BPH studies [54], but was reduced by LLE treatment. Thus, the concurrent reduction in 5α-reductase type 2 and aromatase expression suggests that LLE may influence intraprostatic steroid metabolism.

Western blot analysis was limited to the 200 mg/kg/day group because the molecular analysis was intended to explore mechanisms underlying the anti-hyperplastic effect rather than to establish a molecular dose–response relationship. This dose was selected because it significantly reduced the total prostate index as well as the ventral and dorsolateral prostate indices.

Several constituents of *L. lucidus* may potentially contribute to the anti-hyperplastic effects of LLE. Oleanolic acid and ursolic acid have been isolated from *L. lucidus* leaves [55] and have demonstrated anti-hyperplastic effects in testosterone-induced BPH models, including reductions in prostate enlargement, DHT levels, or 5α-reductase type 2 expression [56,57]. Rosmarinic acid, quantified as a marker compound in LLE in the present study, has also been proposed by molecular docking as a potential modulator of the androgen receptor and steroid 5α-reductase type 2 [58], although this computational prediction requires experimental validation. However, whether these compounds actually contribute to the anti-hyperplastic effects of LLE remains to be established. Bioactivity-guided fractionation and pharmacological evaluation of isolated compounds will therefore be needed to identify the active constituents and clarify their molecular targets.

Apoptotic signaling was also explored because a previous study reported that *L. lucidus* can modulate apoptosis-related pathways [59]. Bax and Bcl-2 are key opposing regulators of the mitochondrial apoptotic pathway, with Bax promoting and Bcl-2 inhibiting apoptosis. However, LLE did not significantly alter Bax, Bcl-2, or the Bax/Bcl-2 ratio in the ventral prostate. These findings do not support a major role for the Bax/Bcl-2-mediated mitochondrial apoptotic pathway under the present experimental conditions. Nevertheless, because apoptosis was assessed only by Bax and Bcl-2 expression, other apoptotic mechanisms cannot be excluded. Additional assessments, including caspase activation and TUNEL staining, would be needed to more comprehensively evaluate the involvement of apoptosis.

Several limitations should be considered when interpreting the present findings. First, the TP-induced BPH model does not reproduce the gradual, age-related hormonal alterations or the full pathological complexity of human BPH. Spontaneous age-related prostatic hyperplasia in dogs and certain nonhuman primates may more closely resemble aspects of human disease, although their use is limited by practical and economic constraints [33]. The present findings should therefore be regarded as initial preclinical evidence obtained under controlled androgen-stimulated conditions and require further validation in complementary models. Second, the mechanistic investigation was focused primarily on androgen metabolism. Intraprostatic androgen concentrations and the enzymatic activities of 5α-reductase and aromatase were not directly measured, and AR signaling was not examined. Therefore, the present findings support an association between LLE treatment and changes in androgen metabolism-related parameters but do not establish direct inhibition of intraprostatic androgen conversion or AR signaling. Further studies evaluating these parameters would help clarify the underlying mechanism.

From a translational perspective, the 200 mg/kg/day dose that produced significant anti-hyperplastic effects corresponds to approximately 20 g/day of crude herbal material for a 60-kg adult. Lycopi Herba has a long history of medicinal use in East Asia and is included in the official pharmacopoeias of several East Asian countries; a dose of 20 g/day has also been documented in a traditional prescription [60]. Although no apparent adverse effects were observed under the present experimental conditions, comprehensive systemic safety was not assessed. Furthermore, body surface area-based conversion is approximate and does not account for interspecies differences in pharmacokinetics. Therefore, further pharmacokinetic and safety studies are warranted to support clinical translation.

## 4. Materials and Methods

### 4.1. Preparation of LLE

Lycopi Herba, the aerial part of *L. lucidus*, was purchased from Dongwoodang Pharmacy Co., Ltd. (Yeongcheon, Republic of Korea). The plant material was authenticated by Prof. Jungbin Song (Kyung Hee University, Seoul, Republic of Korea), and a voucher specimen (No. 23091902) was deposited in the herbarium of the College of Korean Medicine, Kyung Hee University, Seoul, Republic of Korea. The dried herb was extracted with 10 volumes of 70% ethanol by reflux extraction at 80 °C for 3 h. The extract was filtered using filter paper (Whatman™ grade 2, Cytiva, Marlborough, MA, USA) and concentrated at 45 °C using a rotary vacuum evaporator (N-1200A, EYELA, Tokyo, Japan). The concentrate was pre-frozen at −45 °C and lyophilized using a freeze dryer (FDU-1200, EYELA, Tokyo, Japan). The obtained ethanol extract of *L. lucidus* (LLE) had a final yield of 9.84%. The extraction yield showed limited batch-to-batch variability (four batches; RSD, 5.20%).

### 4.2. HPLC Analysis of LLE

LLE was analyzed by HPLC coupled with diode array detection for the quantification of rosmarinic acid. Based on previous reports of its identification and quantification in *L. lucidus* [61,62,63], rosmarinic acid was selected as a marker compound, and an analytical reference standard with a purity of 98.5% was obtained from Sigma-Aldrich (St. Louis, MO, USA). The analysis was performed using an Agilent 1260 Infinity III HPLC system (Agilent Technologies, Santa Clara, CA, USA) equipped with a G7111A quaternary pump, a G7129A vialsampler, and a G7115A diode array detector. HPLC-grade methanol and acetonitrile were obtained from Duksan Pure Chemicals Co., Ltd. (Ansan, Republic of Korea), and trifluoroacetic acid was purchased from Daejung Chemicals & Metals Co., Ltd. (Siheung, Republic of Korea).

LLE (10 mg) was mixed with 1 mL of methanol, ultrasonically extracted for 5 min, and filtered through a 0.2 µm polytetrafluoroethylene syringe filter. A stock solution of rosmarinic acid was prepared in methanol at 2 mg/mL and serially diluted to obtain five calibration standards at concentrations of 0.0625, 0.125, 0.25, 0.5, and 1 mg/mL.

Chromatographic separation was performed at room temperature using a SunFire C18 column (5 µm, 4.6 × 250 mm; Waters, Milford, MA, USA). The mobile phase consisted of 0.1% trifluoroacetic acid in water (solvent A) and acetonitrile (solvent B). Solvent B was maintained at 23% for 20 min, increased to 100% over 5 min, and maintained at 100% for 10 min. The flow rate was 1.0 mL/min, and the injection volume was 10 µL. Rosmarinic acid was detected at 330 nm. Rosmarinic acid in LLE was identified by comparison of its retention time and UV absorption spectrum with those of the reference standard. Data acquisition and processing were performed using OpenLab CDS software (version 3.7; Agilent Technologies). Rosmarinic acid was quantified by linear regression using a five-point calibration curve, and the standard and sample solutions were analyzed in triplicate.

### 4.3. Experimental Animals

Ten-week-old male Sprague–Dawley rats were obtained from Koatech (Pyeongtaek, Republic of Korea). Animals were acclimated for one week before the experiment and maintained under controlled conditions (temperature 23 ± 1 °C, humidity 50 ± 10%, 12 h light/dark cycle) with free access to food and water. All animal procedures were conducted in accordance with the Standard Operating Procedures of the Institutional Animal Care and Use Committee (IACUC) of Kyung Hee University, and the study protocol was approved by the IACUC of Kyung Hee University (Approval No. KHSASP-23-130) on 12 April 2023.

### 4.4. Induction of BPH and Administration of LLE

After one week of acclimation, forty rats were randomly allocated to five groups based on body weight to minimize baseline differences among the groups: sham (*n* = 6), control (*n* = 9), finasteride (1 mg/kg/day, *n* = 7), LLE 100 mg/kg/day (*n* = 9), and LLE 200 mg/kg/day (*n* = 9). Group sizes were determined based on our previous studies using this model [47,64], sample sizes used in comparable studies [65,66], and the reduction principle of the 3Rs to minimize unnecessary animal use. A formal power calculation was not conducted before the experiment. No animals were excluded after randomization, and all randomized animals completed the experimental protocol and were included in the analyses.

All groups except the sham group underwent bilateral orchiectomy under isoflurane anesthesia according to the method described by Brown (2008) [67]. After a midline abdominal incision, the testes, epididymides, and epididymal fat pads were removed, and the incision was sutured. Animals were allowed to recover for one week. The sham group underwent the same surgical procedure without removal of any tissues. After recovery, BPH was induced by subcutaneous injection of TP (3 mg/kg; TCI, Tokyo, Japan) dissolved in olive oil once daily for 28 days in all groups except the sham group. The sham group received olive oil alone. The injection volume was 1 mL/kg body weight. During the sample period, LLE or finasteride (Proscar^®^, Organon, Jersey City, NJ, USA) was administered orally once daily for 28 days. The sham and control groups received distilled water. The oral administration volume was 10 mL/kg body weight.

### 4.5. Blood Collection and Prostate Dissection

Animals were fasted overnight before sacrifice, and fasting body weight was recorded. Blood was collected from the inferior vena cava under isoflurane anesthesia. The ventral, dorsolateral, and anterior lobes of the prostate were excised and weighed separately. The total prostate weight was calculated as the sum of the ventral, dorsolateral, and anterior prostate weights. Blood samples were placed in serum separator tubes (BD Vacutainer^®^, BD, Franklin Lakes, NJ, USA) and kept on ice. Serum was obtained by centrifugation at 3500 rpm for 10 min at 4 °C using a centrifuge (5810R, Eppendorf, Hamburg, Germany) and stored at −80 °C until analysis. For histological analysis, ventral prostate tissues were fixed in 10% formalin at 4 °C. For Western blot analysis, ventral prostate tissues were snap-frozen in liquid nitrogen immediately after excision and stored at −80 °C. The prostate index was calculated as prostate weight (mg) per 100 g fasting body weight. The inhibition rate (%) was calculated using the formula: (Control–Treatment)/(Control–Sham) × 100.

### 4.6. Histological Analysis

#### 4.6.1. H&E Staining

Formalin-fixed prostate tissues were dehydrated, embedded in paraffin, and sectioned at 4 µm thickness in the transverse plane at the central region of the prostate. Sections were stained with H&E and examined using a light microscope (Eclipse Ci-L, Nikon Instruments Inc., Melville, NY, USA).

#### 4.6.2. Histological Scoring

Histological changes were evaluated using a semi-quantitative scoring system based on the method proposed by Scolnik et al. (1994) [43] and modified by Golomb et al. (1998) [44]. This scoring system was developed to assess hyperplastic changes in the ventral prostate of rats and reflects morphological alterations in the acinus, lumen, interacinar space, and stroma. This scoring approach has been applied in several rat models of prostatic hyperplasia, including testosterone-induced models [45,46,47]. Each parameter was scored according to severity and distribution, with higher scores indicating more severe hyperplasia and pathological changes. All slides were independently evaluated by two investigators who were blinded to the treatment groups and applied the same predefined scoring criteria. For each animal, five random fields were examined at low magnification (100×) and five at high magnification (400×), and the mean of the total scores assigned by the two investigators was used for statistical analysis.

#### 4.6.3. Measurement of Epithelial Thickness

As a quantitative morphometric assessment, glandular epithelial thickness was measured as the perpendicular distance from the basement membrane to the luminal surface of the prostatic epithelium. Measurements were performed on images captured at 100× magnification using ImageJ software (ver. 1.54a, NIH, Bethesda, MD, USA). Five randomly selected regions per animal were measured, and the mean value was used for statistical analysis.

### 4.7. Measurement of Serum Testosterone and DHT

Serum testosterone and DHT concentrations were measured using competitive enzyme immunoassay kits (CSB-E05100r, CSB-E07879r, Cusabio, Houston, TX, USA) according to the manufacturer’s instructions. All serum samples and standards were equilibrated to room temperature before analysis and measured in duplicate. Absorbance was measured at 450 nm with a correction wavelength of 600 nm using a microplate spectrophotometer (BioTek Epoch 2, Agilent, Santa Clara, CA, USA). Concentrations were calculated using a 4-parameter logistic standard curve.

### 4.8. Western Blot Analysis

Ventral prostate tissues were homogenized in RIPA buffer (Thermo Fisher Scientific, Waltham, MA, USA) using a bead mill homogenizer (Bead Ruptor Elite, OMNI International, Kennesaw, GA, USA). Protein concentration was determined using a BCA protein assay kit (Thermo Fisher Scientific, Waltham, MA, USA). Samples were mixed with Laemmli sample buffer containing 5% β-mercaptoethanol. Equal amounts of protein (50 µg) were separated by sodium dodecyl sulfate–polyacrylamide gel electrophoresis on 4–20% gradient gels (Bio-Rad Laboratories, Hercules, CA, USA) at 150 V for 60 min and transferred to polyvinylidene fluoride membranes (Bio-Rad Laboratories, Hercules, CA, USA) at 100 V for 60 min.

Membranes were blocked with 5% skim milk in Tris-buffered saline containing 0.1% Tween 20 (TBST) for 120 min at room temperature and incubated overnight at 4 °C with primary antibodies against 5α-reductase type 2 (1:2000, MA5-37985, Thermo Fisher Scientific, MA, USA), aromatase (1:1000, A2161, ABclonal Technology, Woburn, MA, USA), Bax (1:200, sc-7480), Bcl-2 (1:200, sc-7382), and β-actin (1:1000, sc-47778) (Santa Cruz Biotechnology, Dallas, TX, USA). After washing with TBST, membranes were incubated with horseradish peroxidase-conjugated secondary antibodies for 1 h at room temperature, including goat anti-mouse IgG (31430, Thermo Fisher Scientific, Waltham, MA, USA) and goat anti-rabbit IgG (G-21234, Thermo Fisher Scientific, Waltham, MA, USA; AS014, ABclonal Technology, Woburn, MA, USA).

Protein bands were detected using an enhanced chemiluminescence substrate (Bionics, Seoul, Republic of Korea) and visualized with a ChemiDoc™ MP Imaging System (Bio-Rad Laboratories, Hercules, CA, USA). Band intensities were quantified using Image Lab software (ver. 6.1, Bio-Rad Laboratories, Hercules, CA, USA) and normalized to the β-actin signal.

### 4.9. Statistical Analysis

Data are expressed as mean ± standard deviation (SD). For continuous variables, normality and homogeneity of variance were assessed using the Shapiro–Wilk and Brown–Forsythe tests, respectively. Data meeting both assumptions were analyzed by ordinary one-way analysis of variance (ANOVA) with Dunnett’s test; data with unequal variances were analyzed by Welch’s one-way ANOVA with Dunnett’s T3 test; and non-normally distributed data were analyzed by the Kruskal–Wallis test with Dunn’s test. Histological scores were analyzed by the Kruskal–Wallis test with Dunn’s test. Analyses were performed using GraphPad Prism version 8.4.2 (GraphPad Software, San Diego, CA, USA).

In accordance with ARRIVE 2.0 reporting recommendations, effect sizes for the total prostate index, a key efficacy measure, were estimated using Hedges’ *g* with corresponding 95% confidence intervals. Hedges’ *g* was calculated for LLE 200 mg/kg and finasteride versus the control group, as well as between the two treatments. Hedges’ *g* was selected because it provides a small-sample bias-corrected estimate of the standardized mean difference [68]. Analyses were performed using R version 4.6.1 (R Foundation for Statistical Computing, Vienna, Austria) and the effectsize package version 1.0.3 [69].

## 5. Conclusions

Taken together, the present study provides experimental evidence that *L. lucidus* extract attenuates TP-induced prostatic hyperplasia and that these effects are associated with changes in androgen metabolism-related parameters, including the serum DHT/testosterone ratio and prostatic expression of 5α-reductase type 2 and aromatase. These findings provide a basis for further investigation of *L. lucidus* extract in BPH.

## Figures and Tables

**Table 1:** Body weights and body weight gain during the experimental period.

Group	Surgery	Day 1	Day 29	Body Weight Gain (Day 29–Day 1)
Sham	344.4 ± 7.1	366.4 ± 8.1 *	418.8 ± 13.9 ***	52.4 ± 10.5 ***
Control	345.7 ± 7.8	348.2 ± 11.2	363.2 ± 16.5	15.0 ± 19.0
Finasteride 1 mg/kg	349.5 ± 14.0	354.1 ± 16.5	381.7 ± 14.1	27.6 ± 6.5
LLE 100 mg/kg	346.0 ± 10.0	345.0 ± 13.5	366.9 ± 12.5	21.9 ± 5.7
LLE 200 mg/kg	335.9 ± 11.6	331.8 ± 13.1	353.4 ± 16.8	21.6 ± 11.3

Data are expressed as the mean ± SD in grams (g). Surgery refers to the day of sham operation or castration. Day 1 and Day 29 indicate the first treatment day and sacrifice day, respectively; body weight gain was calculated as Day 29 minus Day 1 body weight. LLE, *Lycopus lucidus* extract. * *p* < 0.05, *** *p* < 0.001 vs. Control.

**Table 2:** Histological scoring analysis of the ventral prostate in testosterone propionate–induced benign prostatic hyperplasia rats.

Variable (Score)	Sham	Control	Fina	LLE 100 mg/kg	LLE 200 mg/kg
**Low magnification (100** **×** **)**					
Luminal shape	2.00 (1.10) ***	4.44 (0.73)	2.71 (0.76) **	3.00 (1.22) *	2.78 (0.67) **
Acinar shape	1.00 (0.00) ***	4.33 (1.00)	3.00 (1.15)	1.89 (1.05) **	2.56 (0.88)
Interacinar space	1.00 (0.00)	2.33 (2.00)	1.57 (1.51)	1.00 (0.00)	1.00 (0.00)
Stroma	1.00 (0.00) **	3.22 (1.20)	1.86 (1.07)	1.00 (0.00) ***	1.67 (1.00) *
**High magnification (400** **×** **)**					
Epithelial shape	1.07 (0.16) ***	2.51 (0.59)	1.80 (0.52)	1.40 (0.35) *	1.40 (0.49) **
Number of layers	1.47 (0.39) ***	2.78 (0.49)	1.97 (0.65)	1.89 (0.27) *	1.71 (0.56) **
Layer distribution	1.00 (1.55) *	3.56 (1.67)	2.14 (1.46)	2.89 (1.27)	2.78 (1.79)
Polarity	1.67 (1.03) *	3.00 (0.00)	2.43 (0.98)	2.33 (1.00)	2.56 (0.88)
Epithelial piling	1.00 (1.55)	2.67 (1.00)	2.14 (1.46)	2.00 (1.50)	2.00 (1.50)
Budding into stroma	1.67 (2.58)	2.78 (2.64)	0.71 (1.89)	1.67 (2.50)	2.78 (2.64)
Periacinar epithelial cluster	0.00 (0.00)	1.33 (1.58)	0.86 (1.46)	1.33 (1.58)	0.33 (1.00)
Isolated epithelial cluster	0.00 (0.00)	1.11 (2.20)	0.71 (1.89)	0.56 (1.67)	0.56 (1.67)
Lesion distribution	1.33 (2.07) **	6.44 (1.94)	2.29 (2.14) *	2.67 (2.83) *	2.67 (3.46) *
Nuclear shape	1.00 (0.00) ***	5.00 (0.00)	2.71 (2.14)	3.22 (2.11)	3.67 (2.00)
Nuclear size	2.67 (1.03)	3.11 (1.05)	2.86 (1.07)	2.00 (0.00)	2.67 (1.00)
Mitoses	1.33 (1.03)	2.33 (2.18)	1.14 (1.07)	1.67 (1.58)	0.89 (1.05)
Basement membrane	1.67 (1.63)	3.22 (2.11)	2.71 (2.14)	3.22 (2.11)	1.89 (1.76)
Membrane thickness	1.00 (0.00) *	3.22 (2.11)	1.57 (1.51)	1.00 (0.00) **	1.44 (1.33)
**Total score**	21.87 (7.55) ***	**57.40** **(10.25)**	**35.20** (11.05) *	**34.73** (8.85) *	**35.33** (13.03) *

* *p* < 0.05, ** *p* < 0.01, and *** *p* < 0.001 vs. Control; Fina, finasteride 1 mg/kg; LLE, *Lycopus lucidus* extract. Values are presented as mean (SD).

## Data Availability

The data presented in this study are available on request from the corresponding author due to pending patent applications.

## Abbreviations

The following abbreviations are used in this manuscript:

BPH	Benign Prostatic Hyperplasia
DHT	Dihydrotestosterone
H&E	Hematoxylin–Eosin
HPLC	High-Performance Liquid Chromatography
IACUC	Institutional Animal Care and Use Committee
LLE	*Lycopus lucidus* extract
TBST	Tris-buffered saline containing 0.1% Tween 20
TP	Testosterone propionate
